# Identification of selection and inhibition components in a Go/NoGo task from EEG spectra using a machine learning classifier

**DOI:** 10.1002/brb3.1902

**Published:** 2020-10-19

**Authors:** Bambi L. DeLaRosa, Jeffrey S. Spence, Michael A. Motes, Wing To, Sven Vanneste, Michael A. Kraut, John Hart

**Affiliations:** ^1^ School of Brain and Behavioral Sciences The University of Texas at Dallas Dallas TX USA; ^2^ Center for BrainHealth The University of Texas at Dallas Dallas TX USA; ^3^ Callier Center – Dallas The University of Texas at Dallas TX USA; ^4^ Department of Radiology The Johns Hopkins University School of Medicine Baltimore MD USA

**Keywords:** EEG, Go/NoGo, machine learning, time frequency

## Abstract

**Introduction:**

Prior Go/NoGo studies have localized specific regions and EEG spectra for which traditional approaches have distinguished between Go and NoGo conditions. A more detailed characterization of the spatial distribution and timing of the synchronization of frequency bands would contribute substantially to the clarification of neural mechanisms that underlie performance of the Go/NoGo task.

**Methods:**

The present study used a machine learning approach to learn the features that distinguish between ERSPs involved in selection and inhibition in a Go/NoGo task. A single‐layer neural network classifier was used to predict task conditions for each subject to characterize ERSPs associated with Go versus NoGo trials.

**Results:**

The final classifier accurately identified individual task conditions at an overall rate of 92%, estimated by fivefold cross‐validation. The detailed accounting of EEG time–frequency patterns localized to brain regions (i.e., thalamus, pre‐SMA, orbitofrontal cortex, and superior parietal cortex) corroborates and also elaborates upon previous findings from fMRI and EEG studies, and expands the information about EEG power changes in multiple frequency bands (i.e., primarily theta power increase, alpha decreases, and beta increases and decreases) within these regions underlying the selection and inhibition processes engaged in the Go and NoGo trials.

**Conclusion:**

This time–frequency‐based classifier extends previous spatiotemporal findings and provides information about neural mechanisms underlying selection and inhibition processes engaged in Go and NoGo trials, respectively. This neural network classifier can be used to assess time–frequency patterns from an individual subject and thus may offer insight into therapeutic uses of neuromodulation in neural dysfunction.

## Graphical abstract

We used a relatively large dataset of EEG time–frequency measures for the Go/NoGo task to develop a machine learning based classifier of successful selection and inhibition trials. This neural network classifier accurately identified individual task conditions at an overall rate of 92%, estimated by fivefold cross‐validation. The signatures of the classifier not only replicated the previous main findings of the Neural Hybrid model for selection and inhibition processes in location and time–frequency correlates, but extended these findings with additional signatures not previously isolated.

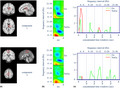

## 1 INTRODUCTION

Go/NoGo tasks have been used in cognitive neuroscience to explore brain mechanisms underlying inhibitory control, selection, and, more broadly, cognitive control (e.g., see [Criaud & Boulinguez, 2015]). Cognitive control deficits and inhibitory dysfunction, in particular, have been linked to clinical conditions such as Attention Deficit Disorder (ADD) [Casey et al., 1997], traumatic brain injury (TBI) ([Dockree & Robertson, 2011], and schizophrenia [Carter et al., 2001], and a host of studies have sought to understand the neural mechanisms supporting inhibition and selection. Go/NoGo tasks require responding to designated “Go” stimuli (e.g., a green square) and withholding responding to other designated “NoGo” stimuli (e.g., a red square). The proportion of Go stimuli is typically higher than NoGo stimuli (e.g., 80% Go and 20% NoGo) to establish a biased expectation to respond on any given trial. Go/NoGo task performance then depends on the abilities both to select to respond to Go stimuli as well as inhibit the established prepotent response to NoGo stimuli.

Scalp‐recorded EEG signals consist of mixed signals from various neural sources. The preponderance of electroen cephalography (EEG) research on selection and inhibition in Go/NoGo paradigms has focused on N2 and P3 ERP components. On both Go‐ and NoGo trials, both a negative deflection of the ERP occurring between 250 and 350 ms (i.e., N2), and a positive deflection occurring between 300 and 600 ms (i.e., P3) have been observed, both with midline frontal to parietal distributions and with NoGo waveform amplitudes exceeding that of Go (Bruin & Wijers, 2002; Maguire et al., 2009; Nakata et al., 2004; Nakata et al., 2010). The N2‐P3 components have been shown to relate to inhibitory processes, but the relationships of these components to specific inhibitory functions remain to be fully specified (Huster et al., 2013).

In studies examining EEG oscillatory dynamics in Go/NoGo tasks, frontal potential fluctuations in the theta range (i.e., 4–8 Hz) have been posited to index processes related to selection and inhibition (Kirmizi‐Alsan et al., 2006; Yamanaka & Yamamoto, 2010), with theta‐power accentuated in inhibition compared to selection. In a previous study performed by our group, we found that theta‐power between 200 and 600 ms after the onset of a trial was greater during NoGo than during Go trials and found local maxima for the theta‐power increase over two fronto‐central regions corresponding to frontal pole and pre‐SMA, with theta oscillations being coherent between the two regions for the NoGo condition (Brier et al., 2010). Furthermore, EEG source localization analysis of Go/NoGo data has suggested anterior–posterior distinctions between NoGo and Go signal generators, with NoGo signal generators having a more anterior localization (Bokura et al., 2001). Magneto‐encephalography (MEG) findings also have suggested localization of inhibitory control signals to fronto‐central regions (Sasaki et al., 1993), and midline theta changes in EEG corresponding to medial frontal/anterior cingulate theta fluctuations in MEG (Asada et al., 1999).

Functional MRI studies have provided anatomic localization in Go/NoGo tasks. Functional MRI signal from the pre‐SMA was increased for both Go and NoGo trials (Chiang et al., 2013), suggesting involvement in both response selection and inhibition. Additionally, the pre‐SMA showed greater activation on NoGo than on Go trials, consistent with the time–frequency data, suggesting additional involvement in response inhibition. Inhibition‐related effects (i.e., greater fMRI signal change on NoGo compared to Go trials) also were observed within right tempro‐parietal, right inferior and middle frontal, middle temporal, and precentral and postcentral gyrus regions. Furthermore, in a meta‐analysis of fMRI studies of response inhibition, NoGo stimuli preferentially were found to engage a network including right dorsolateral prefrontal cortex (DLPFC), right inferior frontal gyrus, right inferior parietal lobule (IPL), pre‐SMA, anterior cingulate cortex (ACC), and the insula (Criaud & Boulinguez, 2013).

Lesion studies also have helped delineate essential roles for the frontal pole and the pre‐SMA in selection/inhibition tasks. Lesions of the frontal pole have been shown to increase reaction times substantially in Go/NoGo tasks (Picton et al., 2006), and lesions of pre‐SMA areas have been shown to increase the number of false‐positives to NoGo stimuli (Drewe, 1975; Picton et al., 2006). These lesion‐related findings support the concept that one role the pre‐SMA area plays is in inhibiting the motor action.

An integrated model incorporating functional imaging, electrophysiology and lesion data has been proposed to account for the mechanisms underlying the selection and inhibition processes of item retrieval from semantic memory central to the Go/NoGo task – the Neural Hybrid model (Hart et al., 2013). The time–frequency aspects of the model have shown that theta power for the Go/NoGo task used in the present study increases early in the midline frontal region of the pre‐SMA, which modulates orbitofrontal regions for both the Go and NoGo stimuli, although greater for the NoGo stimuli. Conversely, alpha power was found to decrease in the pre‐SMA region for both the Go and NoGo stimuli (Brier et al., 2010; Hart et al., 2013). Additionally, when stimuli activate an object representation, as required by the Go/NoGo stimuli, there is an increase in low beta power in the pre‐SMA and thalamus (Ferree et al., 2009; Hart et al., 2013; Slotnick et al., 2002). The analytical approached in these previous studies, however, were limited in the ability to identify all regions contributing to Go/NoGo trial task performance, and to determine regional power changes within frequency bands for each conditions, particularly if a frequency band significantly changes multiple times at a given region during a condition.

While there is an accumulating body of data regarding the spatial distribution of frequency‐band specific EEG changes and localization of function via fMRI and lesion studies, there has been less attention paid thus far as to the temporal sequence of these changes. A combination of the spatial and the temporal attributes of the EEG change would contribute substantially to the clarification of neural mechanisms that underlie performance of the Go/NoGo task, and response inhibition more generally. In the present study, we have investigated a Go/NoGo task while recording EEG and evaluating event‐related spectral perturbations (ERSP) (Makeig, 1993) of independent EEG components. We used a machine learning approach to identify the spectral, temporal, and spatial electrophysiological features involved during selection and inhibition. The features learned by the neural network classifier included the identification of the sources of the components, the multiple frequency content of components, and the timing of maximal synchronization or desynchronization of activity that differentiate Go and NoGo trials. The goal was not to determine an optimal classifier amongst multiple established classifier frameworks or creating novel classifiers. Rather, the goal was to construct a classifier that could potentially identify power fluctuations in multiple time–frequency bands contributing to a Go or NoGo response from the same brain region, identifying the temporal order of the ERSPs throughout a response condition, provide temporal envelopes of the duration of an ERSP, and to confirm and/or extend the foundations of the Neural Hybrid model.

## 2 METHODS AND MATERIALS

### 2.1 Subjects

Fifty‐nine subjects (31 M, 28 F) were recruited from the University of Texas at Dallas community via word of mouth and web‐based advertising. All subjects were between the ages of 18 and 35. The mean age of the group was 23.5 (*SD* 4.1). There were 3 African American, 1 American Indian, 16 Asian, 24 Caucasian, 10 Hispanic, and 5 Multiracial participants. The subjects were all college students or graduates with at least 12 years of education.

Subjects were screened, per exclusion criteria, to be free from a history of traumatic brain injury and other significant neurological issues (stroke, seizure disorders, history of high fevers, tumors, or learning disabilities). Exclusion criteria also included left‐handedness, use of alcohol or other controlled substances within 24 hr of EEG administration, and medications other than over‐the‐counter analgesics and oral contraceptives. Two subjects were excluded from the analysis due to corrupted EEG files.

Informed written consent was collected from each subject according to the rules of the Institutional Review Board of The University of Texas at Dallas. This study was conducted according to the Good Clinical Practice Guidelines, the Declaration of Helsinki, and the US Code of Federal Regulations.

### 2.2 Stimuli

We used a basic Go/NoGo paradigm consisting of 160 (80%) ‘Go’ stimuli (a drawing of a specific car) for which the subject was to press a button and 40 (20%) ‘NoGo’ stimuli (a drawing of a specific dog) for which the subject was instructed to withhold a response. The stimuli were presented for 300 ms followed by a fixation point (+) for 1,700 ms. All of the stimuli were black line drawings fitted to a white 600 × 600‐pixel square. The instructions were to press the button for all cars but to do not press the button for anything else. Instructions were given verbally and displayed on the computer screen prior to the task.

### 2.3 EEG recording

Continuous EEG was recorded from a 64‐electrode Neuroscan Quickcap using Neuroscan SynAmps2 amplifiers and Scan 4.3.2 software, with a reference electrode located near the calvarial vertex. Data were sampled at 1 kHz, with electrode impedances typically below 10 kΩ. Additionally, bipolar electrooculographic data were recorded from two electrodes to monitor blinks and eye movements (positioned vertically at the supraorbital ridge and lower outer canthus of the left eye). The continuous EEG data were offline high‐pass filtered at 1 Hz and low‐pass filtered at 50 Hz using a finite impulse response (FIR) filter.

### 2.4 EEG preprocessing

We analyzed the EEG data using scripts developed in our lab that implement functions from EEGLAB version 13.1 [20] running under Matlab 7.11.0. Preprocessing consisted of down‐sampling to 512 Hz, removing data recorded from poorly functioning electrodes, and correcting for stereotyped artifacts including eye blinks, lateral eye movements, muscle, line noise, and heart rate using the runica algorithm (Delorme & Makeig, 2004; Jung et al., 2000), an implementation of the logistic infomax independent component analysis algorithm of Bell & Sejnowski (1,005). Stereotyped artifacts were identified by visual inspection of the spatial and temporal representation of the independent components. Continuous data were then segmented into 3‐s nonoverlapping epochs spanning from 1,000 ms before to 2000 ms after the presentation of the visual stimuli. Epochs containing high amplitude, high‐frequency muscle noise, and other irregular artifacts were removed retaining on average 85 percent of all epochs. Finally, missing electrodes were interpolated and data were rereferenced to the average reference (Junghöfer et al., 2000).

### 2.5 Obtaining independent sources of EEG signals

The cleaned and filtered EEG data were reshaped for purposes of isolating independent sources through Independent Components Analysis (ICA). For each subject and at each channel, the task condition trials were mean centered and concatenated. Each subject's concatenated trials were then scaled to have equal variance. At this stage, all subjects were concatenated to yield a large EEG data frame containing the set of 3‐s trials (−1 to 2 s poststimulus), down‐sampled at 128 Hz, across task conditions and subjects for each channel. We obtained the components from the ICA using the extended infomax option in EEGLAB's runica function and temporarily kept all components for purposes of fitting single dipole scalp projections.

Taking advantage of the fact that independent components are more dipole‐like than the raw channel‐level EEG signals (Delorme et al., 2012), we used EEGLAB's dipfit function (boundary element method) to localize equivalent dipole sources of independent component scalp maps. This procedure allowed us to obtain approximate locations within the brain of the independent component sources themselves. We discarded components that were not isolated to gray matter, and we discarded components whose residual variance was greater than 15% from the fit of the independent component scalp maps to the scalp projections of single equivalent dipoles. The process of attrition left 22 viable independent components and the approximate locations within the brain of their equivalent dipole sources.

### 2.6 Spectral estimation of independent components

Before estimation of the frequency spectra of the derived components from ICA, we isolated the subject‐level data by partitioning each subject's respective segments of the components for both task conditions. In addition, we saved the ICA weights for purposes of projecting individual subjects and their task conditions into the derived component space. This latter step was important for cross‐validation (see details below) in which subjects who were left out of the ICA and subsequent dipole fitting stage could be projected into the appropriate space for later prediction.

Once subject‐level component segments for each condition were isolated, we calculated the frequency spectrum using Matlab's newtimef function in cosine tapered windows across 0.05‐s segments of the trial window. The modulus of the Fourier transform was converted to log scale, and each poststimulus trial segment was baseline‐corrected using the 0.75 s prestimulus onset period as baseline. The frequency range was from 1 Hz to 30 Hz with a frequency resolution of 1 Hz, and the temporal range was from −0.75 s to 1.75 s poststimulus onset with an effective temporal resolution of 0.05 s. Finally, the time–frequency spectra for each task condition were trial averaged for each subject.

At this stage of data processing, the subject‐level arrays for each task condition consisted of log spectra for 22 independent components localized by fitting projections of equivalent dipole sources, and a time–frequency panel covering up to 30 Hz for a 2.5‐s time window around the time of stimulus onset.

### 2.7 Overview of the machine learning approach

Data features, denoted by *X*, were inputs to the prediction classifier, trained to learn the important elements that could classify the task condition. *X* is a *p*‐length vector comprising scaled, log power spectra at localized sources of independent EEG components, average frequency intervals, and 0.8‐s time windows beginning at the stimulus onset for each condition. The output of the prediction model takes values in the set *C* = {Go, NoGo}, which are the task conditions. We proceeded to train a model that would estimate prediction functions *f_k_*(*X*), *k* ∈ *C,* which are condition probabilities given the input features *X*. A single subject's experimental condition was then predicted as *Ĉ*(*X*) = argmax*_k_ f_k_*(*X*), the most probable class, where “^” denotes the estimate derived from the trained prediction. The final prediction model included the appropriate *p*‐length *X*, as well as all model parameters (see details below), that minimized an estimate of test error, defined as the proportion of *C_k_*(*X*) misclassified as *C_l_*(*X*). Model choice and the estimate of its final test error were both obtained through fivefold cross‐validation (CV).

### 2.8 Deriving input features *x* for the prediction classifier

We chose a single‐layer neural network learning approach, with *m* = 1,..., *M* derived units in the “hidden” layer, to classify experimental conditions for single subjects. This classifier has *M* (*p* + 2) + 2 parameters to estimate, with *X* having *j* = 1,..., *p* features. Since this classifier is overparameterized, one of the additional parameters is an *L*_2_ penalty to impose constraints on the other *M* (*p* + 2) + 1 and to prevent overfitting (details below).

To alleviate a significant computational burden, but more importantly for parsimony and interpretive value, we reduced p to a number beyond which we no longer reduced cross‐validation (CV) error. This was accomplished in two ways—fixing the values of time and frequency and finding a suitable subset of components. First, we fixed the time window to be only 0.8 s following stimulus onset, and we averaged frequencies within the intervals 4–8 Hz, 9–10 Hz, 11–12 Hz, 13–20 Hz, and 21–30 Hz. These intervals roughly correspond to theta band, lower and upper alpha band, lower and upper beta band, respectively. Secondly, we ran combinations of components through CV to obtain a subset with minimum CV error. Specifically, we fixed two which we knew from prior literature and experience to be important (components 1 and 5, see Table 1), and we proceeded to add groups of 3–5 components at a time, followed by reductions of 1–2. This process of forward and backward selection, although not nearly exhaustive, relatively quickly allowed us to find a subset with minimum CV error. Adding any single component to this subset or removing any single component from this subset only increased CV error.

**TABLE 1:** Component numbers from the ICA and their approximate locations based on best fits of the component scalp maps to the scalp projections of single equivalent dipoles

Dipole fits for independent components			
Component #	RV	Coordinates	Approximate location
1	0.9%	(9,−14,19)	Right thalamus
5	9.5%	(−1,−24,62)	Left pre‐SMA
6	8.5%	(−5,53,−22)	Left orbitofrontal
13	3.1%	(−24,−75,26)	Left superior parietal (precuneus)
14	4.7%	(−23,−67,17)	Left occipital (cuneus)
15	8.5%	(68,−52,0)	Right middle temporal

Note

The assessment of each fit is given by % residual variance (RV).

Inputs *X* to the neural network classifier require scaling of the features. We accomplished this by centering each subject and component and setting their respective variances equal to 1. Additionally, we adjusted temporally based on reaction times to Go trials. That is, we regressed time to peak (in absolute value) on average reaction times and adjusted time–frequency windows accordingly. We applied the same adjustment to NoGo trials, which implicitly assumes that Go and NoGo reaction times are approximately proportional. This adjustment was fairly minimal, but it significantly reduced CV error.

### 2.9 Model selection

Choosing the final neural network prediction classifier required finding the number, *M*, of derived units in the “hidden” layer, the subset of independent components making up part of the *p*‐length vector *X*, *L*_2_ penalty *λ*, and the model parameter set *θ* = {*α*′, *β*′} which minimizes the penalized cross‐entropy loss function.

$$L\left(\theta \right)=‐\sum_{i=1}^{N}\sum_{k=1}^{2}y_{\mathrm{ik}}\log f_{k}\left(x_{i}\right)+\lambda P\left(\theta \right),$$

where *y_ik_* is an indicator of task condition, $P\left(\theta \right)=\sum_{\mathrm{jm}}\alpha_{\mathrm{jm}}^{2}+\sum_{m}\beta_{m}^{2}$ is a penalty functional, and *λ* is the *L*_2_ parameter (i.e., weight decay parameter) controlling the influence of the penalty when minimizing the loss function, *L*(θ). The greater *λ* is, the more constrained are the size of the parameters *θ* = {*α*′, *β*′} that are implicitly contained in the prediction function *f_k_*(*x_i_*). As noted above, *λ* is an important consideration due to the fact that the classifier is overparameterized.

The issues noted here were implemented as various options in the nnet function of the nnet package for the R statistical computing environment (http://r‐project.org). All parameters and features — M, *θ* = {*α*′, *β*′}, *λ* and *p* — were chosen to minimize CV error.

### 2.10 Fivefold cross‐validation

**FIGURE 1:**
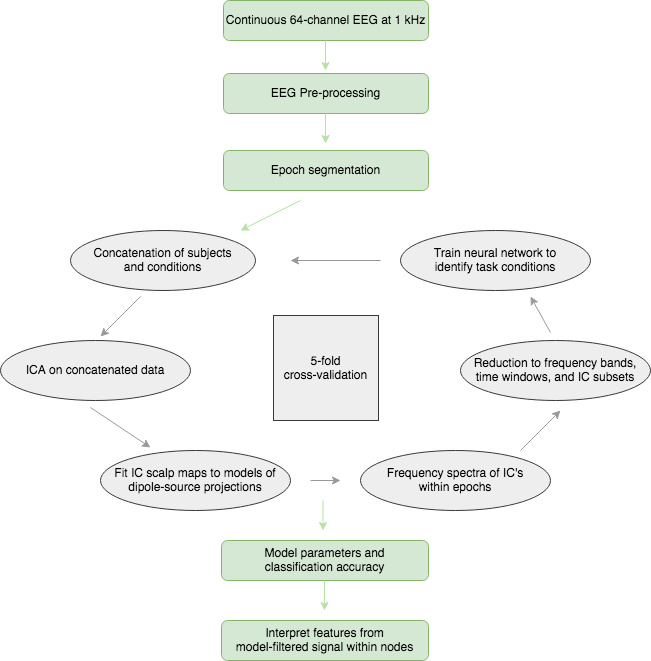
General workflow from raw EEG signal to interpretation using model signatures. The fivefold cross‐validation procedure (in gray) was used to identify important independent components, to determine the penalty parameter, to determine number of nodes in the hidden layer, and to estimate the generalization error (and accuracy) metrics. Final model parameters of the network architecture and the model signatures within nodes (used for interpretation) were calculated from the full data set

Neural network learning models are highly adaptive to training data and will overfit such that predictions using independent input features *X* will generally be poor. We implemented fivefold cross‐validation (CV) to find an optimal model that will generalize well. As noted, CV was used both to find the best classifier and to estimate its test error for generalizability. We emphasize that all aspects of the data processing and classifying stream — from ICA and dipole fitting to the search for feature/parameter sets {*M*, *θ*, *λ*, *p*} — were included in the CV. An overview of the general workflow highlighting these methods is shown in Figure 1.

For this procedure, we randomly partitioned the total sample of subject/conditions, 2 × 57 = 114, into roughly five equal parts. For the *k^th^* partition, or fold comprising 20% of the data, we defined a neural network learning classifier for a particular {*M*, *θ*, *λ*, *p*}‐set, trained on the other 4 parts comprising 80% of the data, then calculated misclassification error (i.e., test error) for the *k^th^* fold using the classifier built without it. This process was repeated five times, once for each of the *k* = 1,..., 5 folds; and the cross‐validation error was estimated by combining the 5 individual estimates. A summary of steps for each candidate classifier was the following:

1. Divide total sample into five folds at random
2. Obtain independent components through ICA using all of the samples *except* those in fold *k*
3. Fit equivalent dipole models to component scalp maps using all of the samples *except* those in fold *k* (Note: component numbers generally did not come out in the same order across the folds, but they were easily matched by finding the closest dipole location within 5 mm)
4. Choose the *p*‐length vector *X*, particularly a subset of independent components, using all of the samples except those in fold *k*
5. Train the neural network learning classifier for each {*M*, *θ*, *λ*, *p*} set using all of the samples except those in fold *k*
6. From the trained classifier obtain the classification *Ĉ*(*X*) and its misclassification error rate for the samples in the hold‐out fold *k*
7. Accumulate errors from all five folds to produce the cross‐validation estimate of test error for the candidate classifier and its associated {*M*, *θ*, *λ*, *p*}‐set.

## 3 RESULTS

### 3.1 Final neural network learning classifier

At the end of classifier selection, we isolated *p* = 240 features comprising input vector *X*: 6 components ×5 frequency intervals ×8 time units between 0 and 0.8 s poststimulus. The frequency intervals were, approximately, theta band, lower and upper alpha band, lower and upper beta band; and the six components are given in Table 1. Approximate locations of these sources are shown, as well as the coordinates of the equivalent dipole having a scalp projection that matches closely with the given component's scalp map.

**FIGURE 2:**
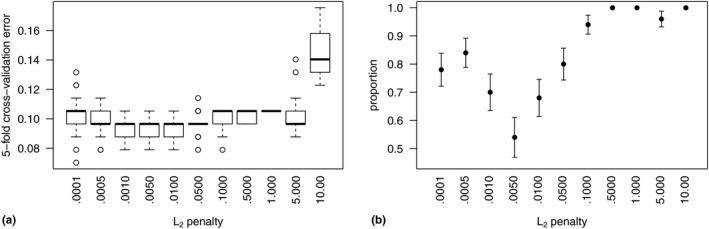
(a) Estimates of the proportion of *C_k_*(*X*) misclassified as *C_l_*(*X*) by fivefold cross‐validation (CV) as a function of the *L*_2_ penalty. Each boxplot is a distribution summary of error estimates from 50 sets of random uniform initialization parameters in the neural network based on *M* = 4 derived units in the single layer. (b) Proportion of the 50 initialization sets that yield a final CV prediction error greater than 0.09. An *L*_2_ penalty equal to 0.005 yields models that are most robust to initial starting values and have a higher proportion of CV prediction error rates equal to that of our final model (0.079)

Cross‐validation chose *M* = 4 units in the hidden layer of the neural network and *λ* = 0.005. Figure 2a shows CV curves as a function of the *L*_2_ penalty for classifiers with *M* = 4 derived units and *p* = 240 features in *X*. The box plots are the CV error distribution summaries of 50 different initialization parameter sets for each value of *λ*. Although the CV error summaries seem relatively flat near *λ* = 0.005, Figure 2b shows that *λ* = 0.005 yields classifiers that are more robust to initialization parameters (i.e., lowest proportion of initialization parameter sets with CV error above 0.09).

**FIGURE 3:**
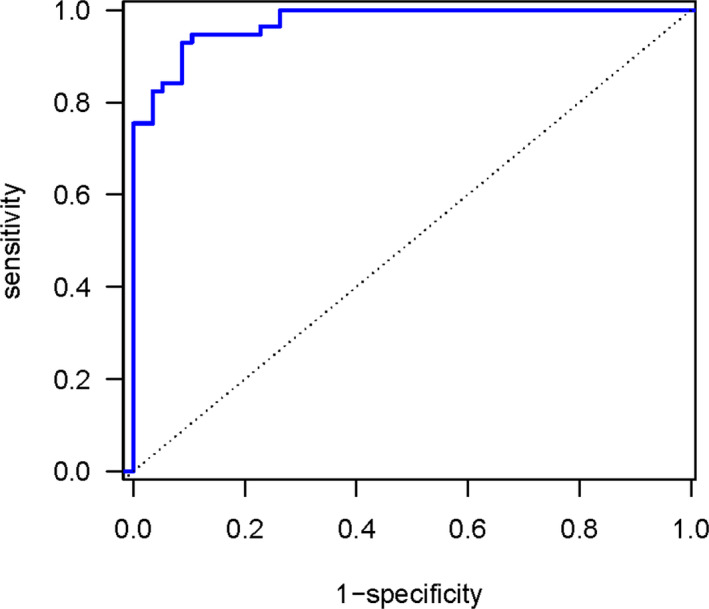
Receiver Operating Characteristics (ROC) curve — sensitivity as a function of the false positive rate (1‐specificity). The area under the curve (AUC) =0.974

The final learning classifier has very good accuracy in predicting task conditions at the subject level. Although the classifier can predict the conditions from the training data with 100% accuracy, the CV error is a better reflection of generalization error when predicting task conditions from independent subjects. These rates are shown in the confusion matrix given in Table 2a. The off‐diagonal entries show the CV error rates for each task condition with an overall error rate of 0.079 (accuracy =0.921, standard error =0.016). Table 2b gives additional accuracy metrics, and Figure 3 shows the ROC curve with an AUC =0.974.

**TABLE 2A:** Confusion matrix, estimated by fivefold cross‐validation, for the neural network learning model of task condition

*C_k_*(*x*)		
*Ĉ_k_*(*x*)	Go	NoGo
Go	0.473	0.052
NoGo	0.026	0.447

Note

*Ĉ_k_*(*x*) is the predicted condition from the model, and *C_k_*(*x*) is the true experimental condition. The overall test error rate is 0.079 (accuracy = 0.921, se = 0.016).

**TABLE 2B:** Additional accuracy metrics for each task condition

	Precision	Recall	f‐score
Go	0.900	0.947	0.923
NoGo	0.944	0.895	0.919

### 3.2 Interpretation of the prediction classifier

**FIGURE 4:**
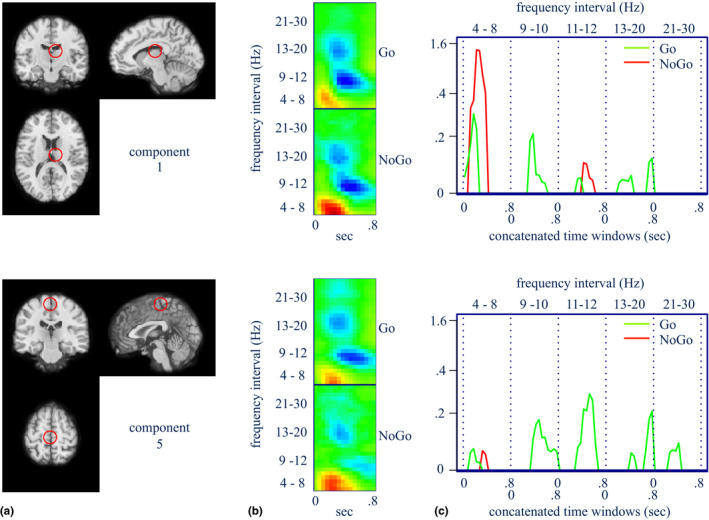
(a) Approximate location of the component source having the best fit of its corresponding scalp map to the scalp projection of a single equivalent dipole. (b) Log power spectra of the independent component for the 0.8‐s time window poststimulus. Red denotes increased and blue denotes decreased spectral power relative to the prestimulus period. (c) Model‐filtered spectral power for frequency intervals and temporal epochs that maximize the prediction functions for each *C_k_*(*X*). These component signatures are viewed here as 1‐D profiles with the 0.8‐s time windows for each frequency interval concatenated along the horizontal axis. (Component 1 is approximately in the right thalamus and component 5 the left pre‐SMA regions)

**FIGURE 5:**
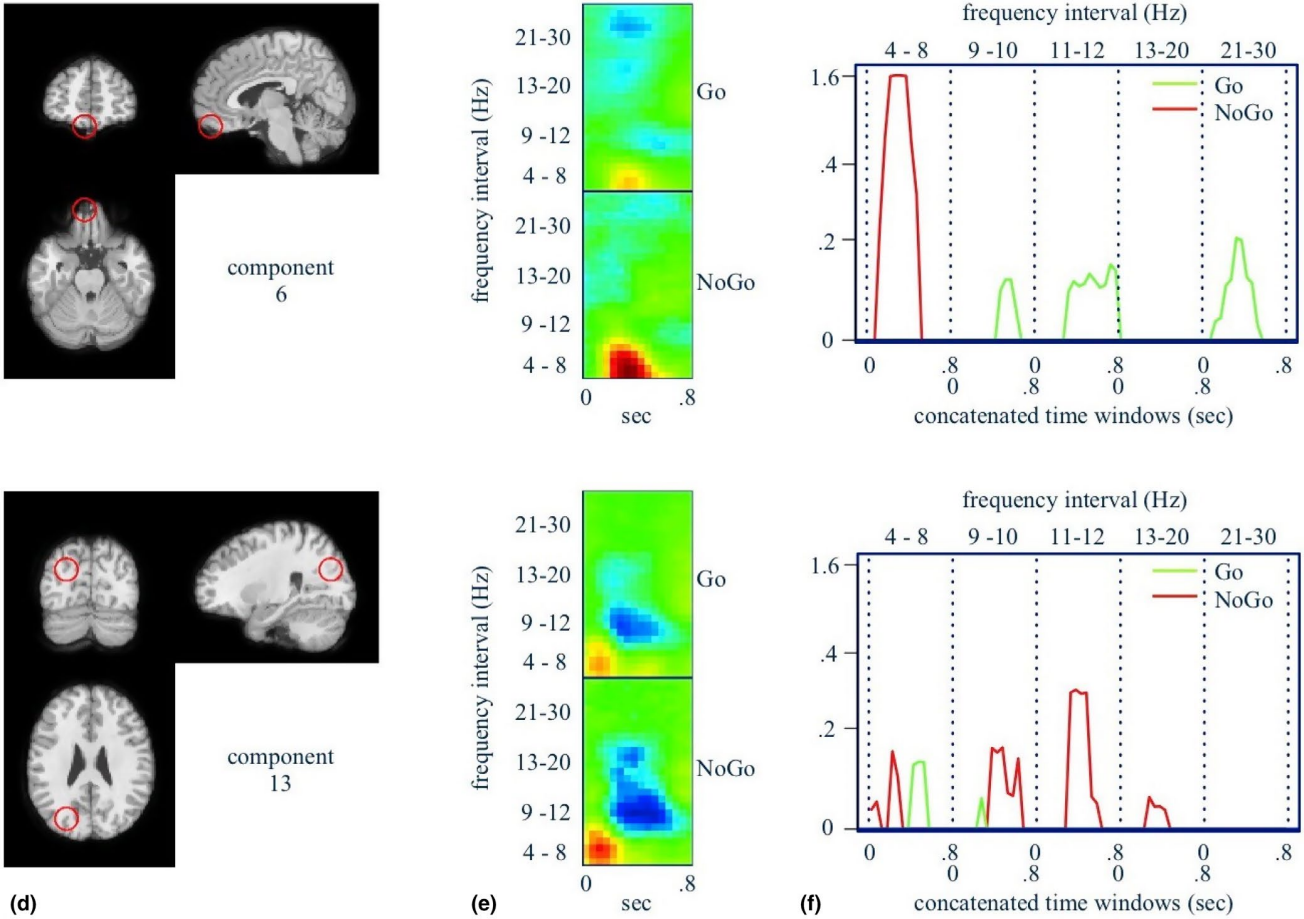
(d) Approximate location of the component source. (e) Log power spectra of the independent component. (f) Component signatures for prediction of *C_k_*(*X*). See Figure 4 for detailed description. (Component 6 is approximately in the left orbitofrontal cortex and component 13 the left superior parietal/precuneus regions)

**FIGURE 6:**
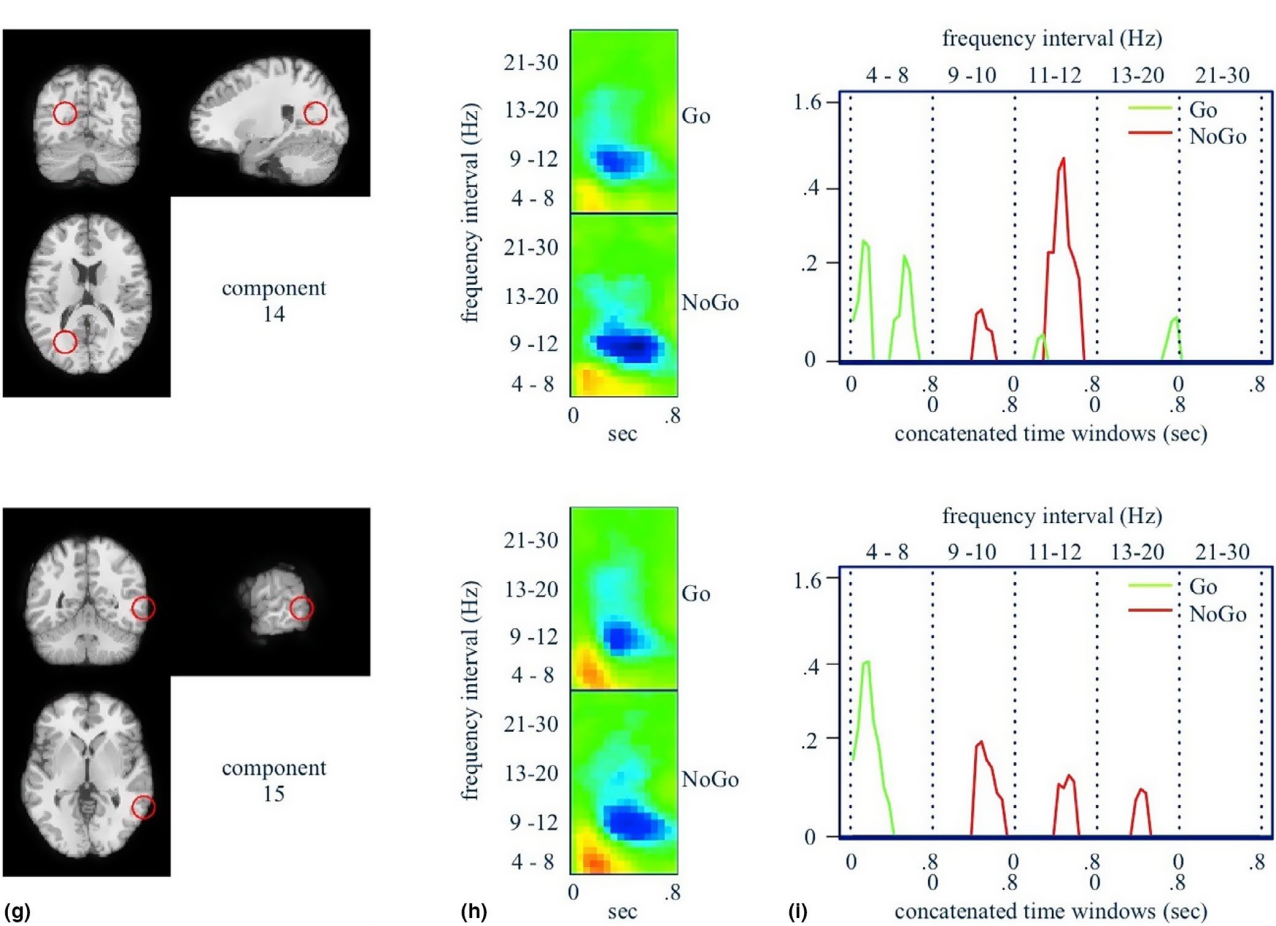
(g) Approximate location of the component source. (h) Log power spectra of the independent component. (i) Component signatures for prediction of *C_k_*(*X*). See Figure 4 for detailed description. (Component 14 is approximately in the left occipital/cuneus and component 15 is the right middle temporal gyrus)

Interpretation of the features in *X* that are essential for accurate task condition predictions requires a more detailed look into the units of the hidden layer of the neural network. These derived units contain the initial information from *X* that contributes to the prediction functions *f_k_*(*X*). For each of the *m* = 1,..., 4 units we used the corresponding classifier parameters in *α*, which are the subset of parameters that relate *X* to each individual unit of the hidden layer, and created a classifier‐filtered version of *X* by the element‐wise product α_jm_ X_j_ *, j* = 1,..., *p*. However, rather than use a classifier‐filtered *X* from a single subject's condition, we chose for purposes of interpretation a “canonical” *X* derived from an average of subjects for whom their task condition predictions both satisfied the criterion that min*_k_*_≠_ *_l_* ${\hat{f}}_{k}$(*X*)/*f* ^ˆ^ *_l_*(*X*) was at least 3. In our sample, 70% of the subjects satisfied this criterion. Figures 4, 5, 6a–i show the main features of the classifier for interpreting the spatial, spectral, and temporal aspects that contribute successful performance for the Go/NoGo task.

Figures 4, 5, 6a,d,g show the locations in the brain of independent components whose scalp maps fit well to scalp projections of equivalent dipoles at those locations (see also Table 1). We interpret these as the approximate locations of the independent EEG sources, given the low %RV of the fits. Figures 4, 5, 6b,e,h show time–frequency maps of an augmented *X* ^∗^ for each task condition (i.e., augmented to include its original frequency resolution). Red denotes increases, and blue denotes decreases, in log spectral power relative to the baseline period. Figures 4, 5, 6c,f,i show the model‐filtered *X* ^∗^ for two of the four derived units in the hidden layer, one whose contribution to *f* ^ˆ^_*k*_(*X* ^∗^) is solely due to the Go condition and one whose contribution to *f* ^ˆ^_*k*_(*X* ^∗^) is solely due to the NoGo condition, shown in green and red, respectively. These are α*_jm_*X*_j_, j* = 1,..., *p*. We have displayed these classifier “signatures” separately by component, and we have reshaped the 2‐D time–frequency panel as a single 1‐D profile, where the 0.8‐s time windows for each frequency interval have been concatenated along the horizontal axis.

All classifier‐filtered peaks are positive because important event‐related increases in log spectral power have positive *α*_jm_ and important event‐related decreases in log spectral power have negative *α_jm_*_._ However, the time–frequency panel reveals the sign of the important peaks. The peaks themselves are statistically significant based on 50,000 nonparametric bootstrap samples of classifier signatures and a Bonferroni threshold given by single‐test levels of 0.05/(2*p*). Notable signatures for NoGo and Go trials are (see also Table 3 for full list):

**TABLE 3:** Listed are the six components that delineate Go and NoGo trials designated by region with the EEG time frequency (theta, lower alpha, upper alpha, lower beta, upper beta) and relative time (early, mid, late) of their peak in the stream of processing for each type of trial

Brain regions	Go	NoGo
Thalamus (right)	Theta (mid) increase	Theta (early) increase
		Lower alpha (mid) decrease
	Upper alpha (mid) decrease	Upper alpha (mid) decrease
		Lower beta (mid) decrease and (late) increase
Left pre‐SMA	Theta (mid) increase	Theta (early) increase
		Lower alpha (mid) decrease
		Upper alpha (late) decrease
		Lower beta (mid) decrease and (late) increase
		Upper beta (mid) decrease
Left orbitofrontal cortex	Theta (mid) increase	
		Lower alpha (mid) decrease
		Upper alpha (mid) decrease
		Upper beta (mid) decrease
Left superior parietal/Precuneus	Theta (early) increase and (mid) increase	Theta (late) increase
	Lower alpha (mid) decrease	
	Upper alpha (mid) decrease	
	Lower beta (mid) decrease	
Left occipital/Cuneus		Theta (early) increase and (late) increase
	Lower alpha (mid) decrease	
	Upper alpha (mid) decrease	
		Lower beta (late) increase
Right middle temporal gyrus		Theta (early) increase
	Lower alpha (mid) decrease	
	Upper alpha (mid) decrease	
	Lower beta (mid) decrease	

#### 3.2.1 NoGo trials

1. Mid time course theta increases in the thalamus, left pre‐SMA, left orbitofrontal cortex, and left superior parietal cortex/precuneus;
2. Mid time course with upper alpha decreases in the thalamus, left superior parietal/precuneus, left occipital/cuneus, and right middle temporal gyrus;
3. Mid time course lower beta decreases in the left superior parietal/precuneus and right middle temporal gyrus;

#### 3.2.2 Go trials

1. Early theta increases in the thalamus, left pre‐SMA, left occipital/cuneus, right middle temporal gyrus, mid time course lower alpha decreases in the thalamus, left pre‐SMA, left orbitofrontal cortex;
2. Mid time course upper alpha decreases in the thalamus, left orbitofrontal cortex;
3. Late lower beta increases in the thalamus, left pre‐SMA, and left occipital lobe/cuneus;
4. Mid time course upper beta decreases in the left pre‐SMA, left orbitofrontal cortex.

## 4 DISCUSSION

In this study, we used a machine learning approach to identify ERSPs that discriminate between selection and inhibition in conventional Go/NoGo task. Our classifier provides a detailed account of time–frequency patterns that differentiate Go and NoGo trials. The purpose of this approach was to characterize a neurophysiological process by a data‐driven method. Neural networks have been very successful at prediction and biomarker development (Acharya et al., 2018; Buettner et al., 2020; Ieracitano et al., 2020), but they have been seldom used for interpretation due to their complexity. We have investigated the nodes of the hidden layer (the derived units) to isolate model‐filtered signatures that mediate task conditions. Thus, we have utilized these signatures for interpretation and to offer insights into the neurophysiological processes themselves. Although theta power increases were expected in thalamus and pre‐SMA from prior group‐level modeling, the machine learning approach brought out a much more complex patterns of processes for this simple task, involving at least six brain regions with previously unknown time–frequency dynamics.

The detailed accounting of EEG time–frequency patterns localized to sources allows for elaboration on previous findings from fMRI and EEG studies by providing more information about EEG power changes in multiple frequency bands per region underlying the selection and inhibition processes engaged in the Go and NoGo trials, respectively (see Table 4). Additionally, the classifier revealed multiple relevant, localized time–frequency changes not previously discussed in earlier selection and inhibition cognitive‐based models (e.g., Neural Hybrid model).

**TABLE 4:** Summary of relevant studies of Go/NoGo and related tasks, techniques of investigation, brain regions localized in the studies, and patterns of associated neural activity

Study	Task	Technique	Localization of task‐related activity	Pattern of task‐related brain activity
Mostofsky & Simmonds, 2008	Go/NoGo	fMRI	Medial frontal	BOLD signal increase for both selection (Go) and inhibition (NoGo)
Kraut et al., 2002	Semantic object recall test	fMRI	Presupplementary motor area (pre‐SMA), caudate, thalamus	BOLD signal increase for selection and retrieval of target
Slotnick et al., 2002	Semantic object recall test	Surface and thalamic electrodes EEG	Thalamus, left occipital	Beta frequency EEG power increase in thalamus and left occipital region for selection and retrieval of target
Brier et al., 2010	Object Go/NoGo	Scalp EEG	pre‐SMA and orbitofrontal pole	Early Theta power EEG power increases in pre‐SMA and orbitofrontal pole for both selection and inhibition (greater for inhibition). Alpha power decreases in pre‐SMA and orbitofrontal pole for selection and inhibition
Hart et al., 2013	Semantic object recall test and object Go/NoGo	fMRI and EEG	pre‐SMA, thalamus, left occipital	Beta frequency EEG power increase for selection and retrieval of target
Chiang et al., 2013	Object Go/NoGo	fMRI	pre‐SMA Right inferior & middle frontal polar, middle temporal, temporoparietal, precentral & postcentral gyri	BOLD signal increase for both selection and inhibition (greater for inhibition) BOLD signal increase for inhibition compared to selection
Criaud & Boulinguez, 2015	Meta‐analysis of Go/Nogo Tasks	fMRI	Right dorsolateral prefrontal, right inferior frontal gyrus, right inferior parietal lobule, pre‐SMA, anterior cingulate, and insula	BOLD signal changes suggest that parietal lobe engaged in decision to act or not in selection or inhibition conditions, attenuates pre‐SMA and motor cortex for inhibition; pre‐SMA engaged in inhibiting motor responses and conflict detection, adjusting response thresholds, and switching from one action plan to another; frontal pole engaged in inhibition
Cooper et al., 2016	Oddball, Go/NoGo, and Switch Tasks	Scalp EEG	Frontoparietal, Midfrontal	Frontoparietal delta EEG power changes– stimulus processing, particularly sensorimotor information; midfrontal theta EEG power changes for all selection and inhibition stimuli related to monitoring response to conflict; central alpha EEG power reduction associated with preparatory switching and anticipatory rule updating for selection

Through our classifier, we identified six components from which neural signals across the EEG spectra, ranging from theta to beta, contributed uniquely to selection and inhibition. Midtrial processing in inhibition involved theta increases in thalamus, pre‐SMA, frontal pole and left parietal/precuneus cortex. Midtrial processing in inhibition also involved (a) decreased upper alpha in thalamus, left parietal/precuneus region, left occipital/cuneus, and right middle temporal gyrus, (b) decreased lower alpha in left parietal/precuneus region, left occipital/cuneus, right middle temporal gyrus, and (c) decreased lower beta in the left parietal/precuneus region and right middle temporal gyrus. Early processing in selection involved (a) theta increases in thalamus, pre‐SMA, left occipital/cuneus region, and right middle temporal gyrus. In selection, midtrial processing involved (a) decreases in lower alpha in thalamus, pre‐SMA, and frontal pole, (b) decreases in upper alpha in thalamus and frontal pole, (c) decreases in lower beta in the thalamus and pre‐SMA, and (d) decreases in upper beta in pre‐SMA, and frontal pole. Finally, in selections, late processing involved (a) theta increases in left parietal/precuneus region, left occipital/cuneus region, (b) upper alpha decrease in pre‐SMA, (c) lower beta increases in thalamus, pre‐SMA, and left occipital/cuneus. These distinctions between Go and NoGo EEG patterns from these sources were shown to be reliable by fivefold cross‐validation, yielding highly accurate prediction rates of normal trial performance in healthy participants.

Our findings are consistent with previous findings showing that medial frontal areas play a role in both response selection and inhibition (Mostofsky & Simmonds, 2008). In fMRI, pre‐SMA and frontal pole (components 5 & 6 in the present modeling) has shown selection‐ and inhibition‐related activation but greater inhibition‐related activation (Chiang et al., 2013; Criaud & Boulinguez, 2015). In other studies, the pre‐SMA showed greater than baseline fMRI signal for both Go and NoGo trials. A meta‐analysis showed that several regions constitute a cognitive control network essential to performing the Go/NoGo task, with the following regions associated with specific cognitive operations, with the parietal lobe with the decision to act or not act in Go‐NoGo conditions and subsequently attenuating activity in pre‐SMA and motor cortex for NoGos, and pre‐SMA with inhibiting motor responses and conflict detection, adjusting response thresholds, and switching from one action plan to another (Criaud & Boulinguez, 2015).

Our results suggest greater neural synchrony in the theta band in inhibition versus selection and possibly reduced alpha synchronization for selection compared to inhibition, underlying the noted activation differences in fMRI. Previous time–frequency studies of the Go/NoGo task showed EEG power changes in the theta and alpha bands, with theta‐band increases during the NoGo trials, and to a lesser degree during the Go trials, over midline frontal EEG electrodes (Brier et al., 2010). Our classifier further characterizes this theta‐band EEG finding and delineates early increases in the pre‐SMA for Go trials relative to NoGo trials and early increases sustained over the trial in the frontal pole for NoGo trials. The temporal and amplitude differences in pre‐SMA might reflect a bias toward selection, possibly related to early template matching on Go trials (Hon et al., 2009; Woolgar et al., 2015) and added matching‐related processing on infrequent NoGo trials. The theta power increase in the frontal pole is consistent with this region's involvement in inhibitory control (Criaud & Boulinguez, 2013).

Our previous EEG time–frequency study also demonstrated declines in alpha power (i.e., desynchronization of regional neural oscillations from baseline) for both Go and NoGo stimuli in midline regions (Brier et al., 2010), with the distribution across both stimulus types/In the Neural Hybrid model, this was interpreted as supporting a common cognitive mechanism, possibly related to evaluation of the stimulus properties. The present classifier localized corresponding midline alpha power changes to pre‐SMA and frontal pole. The classifier, however, shows that the observed alpha desynchronization within these regions was associated with selection and not inhibition. Additionally, however, the classifier reveals inhibition‐related alpha desynchronization within the posterior visual/visual association areas that occurred later in the trial. Alpha‐band oscillations have been imputed to represent inhibitory and timing processes linked to attention suppression and selection (Klimesch, 2012). Across various processing domains, alpha power has been shown to increase in systems targeted for disengagement from a processing task (e.g., the dorsal visual stream when ventral visual stream processing is required [Jokisch & Jensen, 2007]) and decrease in systems targeted for engagement in the task. Within the alpha band, desynchronization or power reductions from baseline are imputed to index a release of “alpha” suppression; whereas, synchronization or power increases are imputed to index an increase in “alpha” suppression. Further desynchronization of lower alpha has been associated with attention allocation and desynchronization of upper alpha has been associated with the Neural Hybrid model semantic retrieval processes (Klimesch, 1999). Thus, the mid‐to‐late increases in lower and upper alpha desynchronization within posterior visual/visual‐association regions on NoGo trials might reflect additional attention and semantic processing taking place after earlier inhibitory control signals from pre‐SMA and frontal pole were initiated. An alternative general account for alpha attenuation in posterior cortical regions is that when midline theta (and likely beta) activation is present, posterior cortical regions encoding representations of items will be characterized by suppressed alpha (Goldman et al., 2002; Jiang et al., 2018). This would be more pronounced for the NoGo trials where disengagement of posterior regions would be indicated if the identified stimulus differs from the predominantly present target, consistent with the Neural Hybrid Model.

The Go responses also differ from the NoGo responses with the presence of lower and upper beta‐band EEG power changes in the processing stream. The late beta EEG power change has been interpreted as mediating final object identification for a decision in the Go trials, and is detected as late beta increases in the model at the pre‐SMA, thalamus and the left occipital region, which are all part of the object identification and name retrieval network (Hart et al., 2013; Slotnick et al., 2002). This network for Go trials is in keeping with the need to clearly designate an item as a Go target by identifying it in semantic memory, which has been identified previously to occur via a pre‐SMA‐thalamic‐cortical representation circuit via beta rhythms (Hart et al., 2013). The network linked by early theta‐band EEG power changes in the pre‐SMA and posterior cortical regions during Go trials are consistent with what has been found in previous studies, with frontal theta being sensitive to increasing amounts of information prior to and upon identification of the stimulus for a Go decision. Such theta EEG signals may reflect communications between posterior cortical regions involved in visual stimulus processing and object identification as noted above. The alpha power changes in the pre‐SMA, orbitofrontal and thalamic regions are proposed to be associated with anticipatory rule updating, motor response (Cooper et al., 2016), and or item search.

The present approach was used to identify the temporal order of spectral perturbations from different general brain regions that would identify Go and NoGo trials with high specificity and sensitivity. The analytical approach was not aimed at identifying the best fitting of many classifiers to find the optimal one for Go and NoGo trials. We acknowledge that other classifiers may exist that perform equally as well as the one used here, including novel components, obtaining optimal estimations of the temporal envelope of time frequencies, and extend or confirm aspects of the previous Neural Hybrid Model. The present analysis adds notably to the Neural Hybrid Model by identifying additional brain regions that are engaged in identifying Go and NoGo stimuli (left superior parietal, right middle temporal, left cuneus) and provides a more detailed account of multiple changes in spectral power during a task within a given region than previous analyses. The analysis confirmed the localizations of the pre‐SMA, orbitofrontal region, and thalamus that had been previously established with fMRI and in the case of the thalamus, intracranial electrode recordings (Chiang et al., 2013; Kraut et al., 2002; Slotnick et al., 2002). Additionally, the classifier chosen allows for comparison of individual subject data to the template developed by our classifier for diagnostic and therapeutic purposes.

Finally, our classifier may have utility in the context of recent advances in nonpharmacological therapeutics in neurological illnesses, chiefly, the use of electromodulation in the treatment of neural dysfunction. A key parameter of neuromodulation is the frequency of the stimulation if alternating current/magnetic fields are involved. Thus, classifiers based on EEG frequency may lead to better insight not only to the dysfunctional circuitry underlying a patient's condition, but also providing insights into the type of electromodulation that may be effective in remediating the dysfunctional state. In addition, time–frequency classifiers allow for making predictions and generating hypotheses concerning nodes and frequencies in the Neural Hybrid model that would be proposed to change with application of electromodulatory stimulation at specific sites. Electrically manipulating the nodes or connections in the brain associated with regions in the model while monitoring accuracy and reaction time will help in refining the model.

As this time–frequency classifier allows for accurate extraction of predictive time–frequency patterns from an individual subject's or potentially a patient's dataset, future plans are to administer this Go/NoGo task to patients who as a consequence of traumatic brain injury now have selection or inhibition defects, and to use the anomalous patterns we detect to guide therapeutic interventions. There have been recent advances in the use of electromodulation as a nonpharmacological intervention for neurological disorders, including treatment of cognitive dysfunction (c.f., Motes et al., 2020; Ulam et al., 2015). Classifiers based on EEG frequency may lead to better insight not only to the dysfunctional circuitry underlying a patient's condition, but also providing insights into the type of electromodulation that may be effective in remediating the dysfunctional state, as the frequency measures derived from the model can guide the key parameters can guide which frequencies may be optimal in the electromodulation techniques of repetitive transcranial magnetic stimulation (rTMS) or High Definition transcranial Alternating Current Stimulation (HD tACS). Additionally, future directions to pursue are applying these techniques at frequencies and brain regions described in the model in normal subjects and determining the effect of this electromodulation on Go/NoGo performance. Through this multi‐pronged approach, we anticipate gaining insight as to how electromodulation affects the neurotypical circuit, and thus how we may remediate a dysfunctional circuit. Finally, applying the techniques we have described to data gathered as normal subjects perform Go/NoGo tasks modified by using more semantically complex stimuli or decision to develop its classifier and compare its frequencies and brain regions to the current one.

## 5 CONCLUSION

The present study extends the Neural Hybrid Model of selection of correct and inhibition of incorrect distractors in semantic memory search by constructing a machine learning classifier to learn the features that distinguish between ERSPs involved in selection and inhibition in a Go/NoGo task. This single‐layer neural network classifier was used to predict accurately identified individual task conditions at an overall rate of 92%, estimated by fivefold cross‐validation. The signatures of the classifier not only replicated the previous main findings of the Neural Hybrid model for selection and inhibition processes in location and time–frequency correlates identified previously by fMRI and EEG studies, but extends these findings by providing more information about neural mechanisms underlying selection and inhibition processes.

## AUTHOR CONTRIBUTION

B.D. was involved in data acquisition, model development, analysis and interpretation of the data, manuscript preparation; J.S. was involved in model development, analysis and interpretation of the data, manuscript preparation; M.M. was involved in experimental design, data acquisition, analysis and interpretation of the data, manuscript preparation; W.T. was involved in experimental design, data acquisition, manuscript preparation; S.V. was involved in experimental design, data acquisition, manuscript preparation; M.K. was involved in experimental design, data acquisition, analysis and interpretation of the data, manuscript preparation; J.H. was involved in experimental design, data acquisition, model development, analysis and interpretation of the data, manuscript preparation.

### Peer Review

The peer review history for this article is available at https://publons.com/publon/10.1002/brb3.1902.

## Data Availability

The data that support the findings of this study are available from the corresponding author upon reasonable request.
